# Challenges of Diagnosis: Periampullary Mass-Induced Obstructive Jaundice in a Young Woman

**DOI:** 10.7759/cureus.61013

**Published:** 2024-05-24

**Authors:** Anusha Gupta, Vijendra Kirnake, Vishal Padwale, Aishwarya Gupta, Sourav Chaturvedi

**Affiliations:** 1 Department of Gastroenterology, Datta Meghe Institute of Higher Education and Research, Wardha, IND; 2 Department of Obstetrics and Gynaecology, Datta Meghe Institute of Higher Education and Research, Wardha, IND; 3 Department of Cardiology, Max Super Speciality Hospital, Delhi, IND

**Keywords:** immunohistochemistry, young adult, adenocarcinoma, obstructive jaundice, pancreaticoduodenectomy, periampullary cancer

## Abstract

Periampullary cancers, which include pancreatic adenocarcinoma, ampullary cancer, distal cholangiocarcinoma, and duodenal cancer, present diagnostic and management challenges due to their aggressive nature and nonspecific symptoms. We describe a case of a female patient, age 20, who had obstructive jaundice brought on by a periampullary tumor. Despite difficulties in diagnosis and treatment, including failed endoscopic retrograde cholangiopancreatography (ERCP), the patient underwent a successful pancreaticoduodenectomy (Whipple's resection), and subsequent immunohistochemistry revealed adenocarcinoma with a mixed immunophenotype expressing duodenal and pancreatic markers. This example emphasizes the significance of taking young patients' periampullary tumors into account, the difficulties in diagnosing them, and the possibility of effective surgical surgery throughout this age range.

## Introduction

Of all periampullary malignancies, adenocarcinoma of the pancreas is the most prevalent, followed by ampullary, duodenal, and distal cholangiocarcinoma [1]. The aggressive nature of these neoplasms can be traced back to delayed diagnosis, with patients often remaining asymptomatic until the disease reaches advanced stages. By the point of diagnosis, more than 50% of patients had already developed distant metastases. The liver is the main site where metastatic lesions occur, with lymph nodes, the peritoneum, the lung, the kidney, and, in rare cases, the skin following.

When dealing with adolescent and young adult patients, it is crucial to consider hereditary syndromes like familial adenomatous polyposis (FAP), Lynch syndrome, Gardner syndrome, and Peutz-Jeghers syndrome as potential differential diagnoses [2]. We document the successful performance of pancreaticoduodenectomy in a 20-year-old female patient diagnosed with periampullary cancer originating from the duodenum. 

## Case presentation

A 20-year-old female patient arrived at the hospital with complaints of generalized weakness, loss of weight and appetite during the previous three months, and yellowish staining of the eyes and urine over the past two months. Her medical and surgical history is clear and free of any past procedures or conditions, indicating a healthy state of being. Upon further history taking, the patient revealed that her mother had succumbed due to colon cancer.

During the physical examination, the patient exhibited icterus. On palpation, she demonstrated a soft, non-tender abdomen. The blood analysis revealed an increased total leukocyte count of 15,000/mm^3^, elevated conjugated bilirubin at 8.0 mg/dL, and abnormal liver function tests, including alanine transaminase (ALT) = 124 IU/L, aspartate aminotransferase (AST) = 130 IU/L, alkaline phosphatase (ALP) = 756 IU/L, indicating obstructive jaundice as enumerated in Table 1. The treatment regimen involved administering intravenous fluids and a seven-day course of piperacillin-tazobactam at a dosage of 4.5 grams every eight hours.

**Table 1:** List of all the investigations performed for the patient along with normal values HBA1c: Hemoglobin A1c; CRP: C-reactive protein; T3: Triiodothyronine; T4: Tetraiodothyronine

S. No.	Investigations	Observed Value	Expected Value
1	Hemoglobin (gm%)	12.4	12-16
2	White Blood Cells (cu.mm)	15000	4000-11000
3	HbA1c	4.4	≤5.6
4	Serum Urea (mg/dL)	24	6-24
5	Serum Creatinine (mg/dL)	0.8	0.7-1.2
6	Serum Sodium (mEq/L)	139	131-145
7	Serum Potassium (mmol/L)	4.4	3.6-5.2
8	Thyroid-Stimulating Hormone (mlU/L)	2.2	0.5-5.0
9	Free T3 (pg/mL)	3.2	2.3-4.1
10	Free T4 (pg/mL)	11.6	9.0-17.0
11	Albumin	3.9 g/dL	3.5-5.0 g/dL
12	Aspartate Aminotransferase	130 IU/L	<50 IU/L
13	Alanine Aminotransferase	124 IU/L	17-59 IU/L
14	Alkaline Phosphatase	756 IU/L	45-147 IU/L
15	Total Bilirubin	8 mg/dL	0.2-1.3 mg/dL
16	International Normalized Ratio	1.58	0.8-1.1
17	Prothrombin Time	20.8	11.9
18	Activated Partial Thromboplastin Time (APTT)	40.2	29.5
19	CRP	10.7 mg/dL	<0.3 mg/dL

Abdominal ultrasound revealed bi-lobar central and peripheral intrahepatic biliary radicle dilatation (IHBRD) along with a dilated common bile duct (CBD) containing a mass in the distal CBD. Contrast-enhanced computed tomography (CECT) abdomen showed minimally enhancing soft tissue density in distal CBD causing upstream dilatation of CBD (16 mm), pancreatic duct (6 mm), double duct sign, and intrahepatic biliary radicle dilatation, suggestive of ? periampullary mass. 

**Figure 1:**
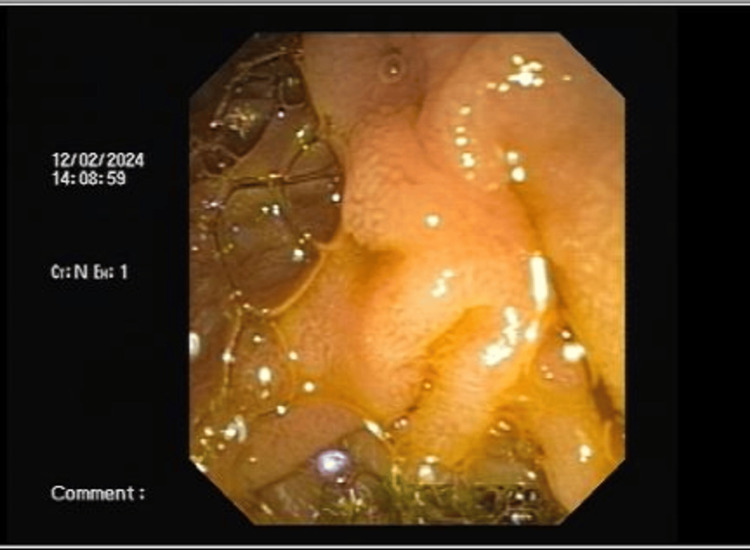
ERCP image showing slightly deformed ampulla ERCP: Endoscopic retrograde cholangiopancreatography

**Figure 2:**
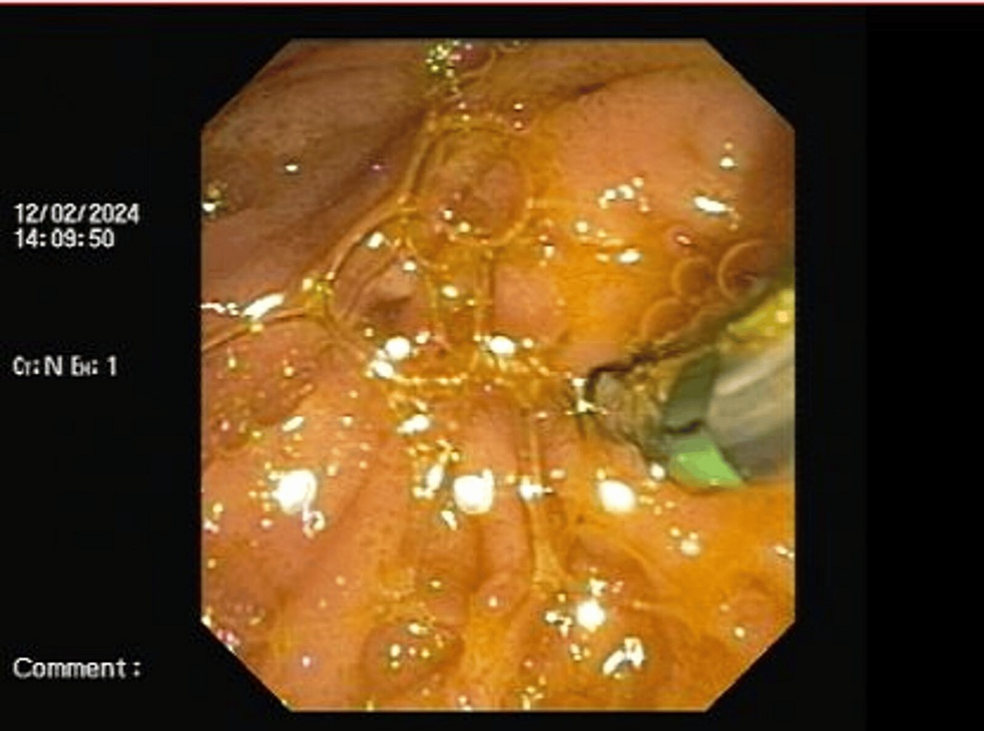
ERCP image showing attempted cannulation of the ampulla ERCP: Endoscopic retrograde cholangiopancreatography

The patient was taken up for endoscopic retrograde cholangiopancreatography (ERCP) but was not successful due to periampullary mass. During ERCP, there was a failure to cannulate the ampulla as shown in Figures 1-2.

**Figure 3:**
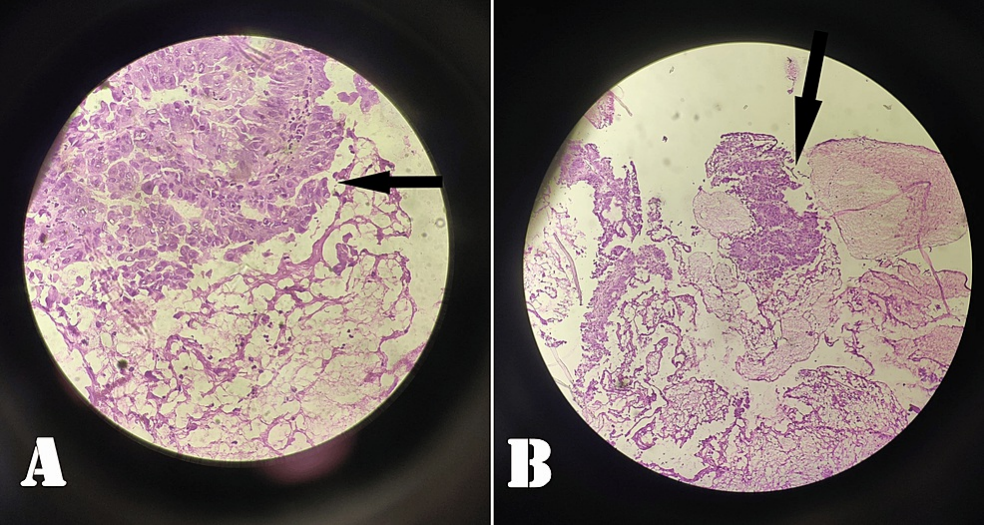
Moderately differentiated adenocarcinoma of the periampullary region (A) and distal CBD (B) CBD: Common bile duct

Multiple biopsies were taken from the ampulla and were sent for histopathological examination (HPE) and a frozen section. The HPE showed moderately differentiated papillary adenocarcinoma of the periampullary area as shown in Figure 3.

The patient underwent percutaneous transhepatic biliary drainage with internal stenting and external drain to decrease the cholangitis. The external drain was subsequently removed. 18-fluoro-deoxyglucose positron emission tomography (FDG PET scan) was done that showed hypermetabolic lesions along the distal CBD and the adjacent second part of the duodenum, measuring 2.1 x 2.7 x 2.6 cm, with minimal dilatation of the CBD, mild IHBRD and pancreatic duct, and surrounding ill-defined stranding - suspicious site of primary malignant etiology needing pathological correlation.

The patient then underwent pancreaticoduodenectomy (Whipple’s resection), which showed ulcero-proliferative growth of size 3.5 x 1.5 x 1.5 cm involving the ampulla of Vater and distal CBD though there was no pancreatic involvement in the resected specimen of the pancreas (6 x 5 x 5 cm), duodenum (22 cm), CBD (2 cm in length), and gall bladder (8 x 4 x 2 cm). The patient was monitored postoperatively and was discharged on postoperative day 25. Immunohistochemistry was performed and is shown in Table 2.

**Table 2:** List of the tumor markers with lead to the conclusion of adenocarcinoma showing mixed immunophenotype expressing duodenal and pancreatic markers CK: Cytokeratin; CDK: Cyclin-dependent kinase; MUC: Mucin

Tumor Markers	Status
CK-7	Positive
CDK-2	Positive
MUC-1	Positive
CK-20	Negative

Post-surgery the patient was stable and symptom-free and was advised for further management in the form of specific targeted chemotherapy; however, the patient refused to undergo chemotherapy.

## Discussion

The periampullary region encompasses a 2 cm radius around the ampulla of Vater. Consequently, this region gives rise to four distinct neoplasms with radiographic features that overlap [3]. They comprise four histologic epithelial types: ampullary, duodenal, pancreatic, and biliary. Given the varying long-term prognoses associated with each of these lesions, it becomes crucial to undergo imaging evaluation for proper characterization of the lesion [4].

More than half of duodenal adenocarcinomas are observed in the periampullary region. Duodenal adenocarcinoma is associated with hereditary syndromes, such as Peutz-Jeghers syndrome, Gardner syndrome, Lynch syndrome, and FAP, establishing these genetic conditions as risk factors for the development of this type of cancer [5].

Patients displaying symptoms of cholangitis should undergo an initial ultrasound examination to assess the hepatobiliary and pancreatic systems [3]. If an ultrasound reveals a dilated duct, CBD, or pancreatic duct, the patient should undergo cholangiography in the form of magnetic resonance cholangiopancreatography (MRCP) or ERCP [6]. MRCP is a non-invasive procedure, whereas ERCP is an invasive procedure and can help identify the site of obstruction.

In the younger population, Whipple's pancreaticoduodenectomy is an uncommon surgery [7]. Consequently, there is very little data on surgical outcomes for this young group, such as delayed gastric emptying, bile leakage, and postoperative pancreatic fistula [8].

However, the patient still needs further management in the form of chemotherapy and genetic studies [9,10].

## Conclusions

Periampullary malignancy is very rare in young adults. Additionally, a thorough evaluation of genetic disorders should be performed on young individuals diagnosed with periampullary cancer. Periampullary adenocarcinomas are rare malignancies with an aggressive course of disease. The tumor's location determines the diagnostic method. A safe method for treating periampullary cancer is Whipple's pancreaticoduodenectomy.

## References

[1] Kumarasamy S, Kaman L, Tandup C, Thakur UK, Savlania A (2022). Periampullary carcinoma in a 13-year-old with microsatellite instability treated successfully with pancreaticoduodenectomy. Cureus.

[2] Jayaramayya K, Balachandar V, Santhy KS (2018). Ampullary carcinoma - a genetic perspective. Mutat Res Rev Mutat Res.

[3] Verma A, Shukla S, Verma N (2015). Diagnosis, preoperative evaluation, and assessment of resectability of pancreatic and periampullary cancer. Indian J Surg.

[4] Wang B, Li D, Zeng D, Wang W, Jiang C (2023). Case report: advanced primary squamous cell carcinoma in the periampullary area with upregulation of programmed cell death-ligand 1 expression and response to sintilimab immunotherapy. Front Immunol.

[5] He J, Ahuja N, Makary MA (2014). 2564 resected periampullary adenocarcinomas at a single institution: trends over three decades. HPB (Oxford).

[6] Lindholm EB, Alkattan AK, Abramson SJ, Price AP, Heaton TE, Balachandran VP, La Quaglia MP (2017). Pancreaticoduodenectomy for pediatric and adolescent pancreatic malignancy: a single-center retrospective analysis. J Pediatr Surg.

[7] Nappo G, Galvanin J, Gentile D (2021). Long-term outcomes after pancreatoduodenectomy for ampullary cancer: the influence of the histological subtypes and comparison with the other periampullary neoplasms. Pancreatology.

[8] Klein F, Jacob D, Bahra M (2014). Prognostic factors for long-term survival in patients with ampullary carcinoma: the results of a 15-year observation period after pancreaticoduodenectomy. HPB Surg.

[9] Duan Z, Zhang Y, Tang Y, Gao R, Bao J, Liang B (2022). Adjuvant therapy for periampullary carcinoma and the significance of histopathological typing: a systematic review. Transl Oncol.

[10] El Nakeeb A, El Sorogy M, Ezzat H (2018). Predictors of long-term survival after pancreaticoduodenectomy for peri-ampullary adenocarcinoma: a retrospective study of 5-year survivors. Hepatobiliary Pancreat Dis Int.

